# A Decade of Ground Deformation in Kunming (China) Revealed by Multi-Temporal Synthetic Aperture Radar Interferometry (InSAR) Technique

**DOI:** 10.3390/s19204425

**Published:** 2019-10-12

**Authors:** Wu Zhu, Wen-Liang Li, Qin Zhang, Yi Yang, Yan Zhang, Wei Qu, Chi-Sheng Wang

**Affiliations:** 1 College of Geology Engineering and Geomatics, Chang’an University, Xi’an 710064, China; 2018226032@chd.edu.cn (W.-L.L.); 2017226007@chd.edu.cn (Y.Z.); quwei@chd.edu.cn (W.Q.); 2 Yunnan Institute of Surveying and Mapping Engineering, Kunming 650033, China; 3 The Guangdong Key Laboratory of Urban Informatics, School of Architecture & Urban Planning, Shenzhen University, Shenzhen 518060, China; wangchisheng@szu.edu.cn; 4 The Key Laboratory of Urban Land Resources Monitoring and Simulation, Ministry of Land and Resources, Beijing 100812, China

**Keywords:** ground deformation, multi-temporal InSAR, Kunming, urbanization

## Abstract

Large-scale urbanization has brought about severe ground subsidence in Kunming (China), threatening the stability of urban infrastructure. Mapping of the spatiotemporal variations of ground deformation is urgently needed, along with summarization of the causes of the subsidence over Kunming with the purpose of disaster prevention and mitigation. In this study, for the first time, a multi-temporal interferometric synthetic aperture radar (InSAR) technique with L-band Advanced Land Observation Satellite (ALOS-1) and X-band Constellation of Small Satellites for Mediterranean basin Observation (COSMO-SkyMed) data was applied to Kunming to derive the time series deformation from 2007 to 2016. The annual deformation velocity revealed two severe subsiding regions in Kunming, with a maximum subsidence of 35 mm/y. The comparison of the deformation between InSAR and leveling showed root-mean-square error (RMSE) values of about 4.5 mm for the L-band and 3.7 mm for the X-band, indicating that our results were reliable. We also found that the L-band illustrated a larger amount of subsidence than the X-band in the tested regions. This difference was mainly caused by the different synthetic aperture radar (SAR)-acquired times and imaging geometries between the L- and X-band SAR images. The vertical time series deformation over two severe subsiding regions presented an approximate linear variation with time, where the cumulative subsidence reached 209 mm during the period of 2007–2016. In view of relevant analyses, we found that the subsidence in Kunming was the result of soft soil consolidation, building load, and groundwater extraction. Our results may provide scientific evidence regarding the sound management of urban construction to mitigate potential damage to infrastructure and the environment.

## 1. Introduction

**Figure 1:**
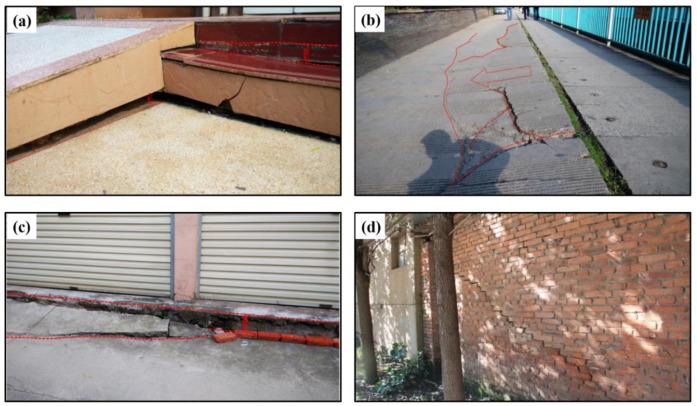
Ground deformation investigated in Kunming. (**a**) the uplift of building steps, (**b**) ground crack, (**c**) ground subsidence, (**d**) wall crack caused by the groud deformation.

Kunming, the capital and largest city of Yunnan province in Southwest China, is located at a longitude of 102°10′–103°40′ east and a latitude of 24°23′–26°22′ north. Situated in the central Yunnan–Guizhou plateau in Southwest China, Kunming is surrounded by the Dian Lake to the south and by mountains on the other three sides [1]. As China’s frontier to Southeast Asia and South Asia, large-scale urban construction has been implemented in Kunming over the past twenty years. During this procedure, a great amount of cultivated land and gardens were occupied by high-rise buildings, roads, and other structures [2]. However, this large-scale urbanization brought about a series of geological hazards, such as landslides, subsidence, and ground fissures. Particularly, ground subsidence became one of the most prominent geological hazards and caused substantial damage to homes, roads, canals, pipelines, and other infrastructure [3]. Figure 1 shows some photos of the effects of subsidence in Kunming, where more than fifty individual damage reports were observed through filed investigations organized in June 2016 [4]. To prevent and mitigate future disasters, it is urgent to characterize and monitor the spatiotemporal variations of ground deformation and summarize the causes of subsidence in Kunming.

Early studies initiated a few years ago using ground-based techniques (e.g., leveling) documented the extent and causes of subsidence in Kunming [5,6]. According to their observations, the subsidence was mainly focused in two regions, where the subsiding rate reached 30 mm/y [5]. Considering local geological conditions and human activities, it was reported that the subsidence in Kunming was mainly caused by groundwater extraction, building load, and natural consolidation of soil [7]. Although these observations provided useful information, they had low spatial resolution, and therefore, more detailed and comprehensive ground deformation was difficult to see. Interferometric synthetic aperture radar (InSAR) has demonstrated its potential for high-density spatial mapping of ground deformation associated with earthquakes [8], volcanoes [9], and other geologic processes. Recent advanced InSAR techniques have improved our understanding of the process of ground deformation, such as the deep learning approach [10], artificial intelligence technique [11], optimal phase unwrapping algorithm [12], signal retrieval for decorrelating targets [13], four-dimensional filtering approach [14], improved synthetic aperture radar (SAR) image coregistration algorithm [15], atmospheric delay correction method [16], and coherent point selection algorithm [17]. Additionally, an increasing amount of SAR satellites have provided a large set of multi-sensor SAR images, which have been employed to reconstruct the spatiotemporal evolution of ground deformation [18]. In this field, current research is focused on three aspects. The first aspect is applying the multi-sensor InSAR to derive the long-term ground deformation, such as in Tehran (Iran) [19], Xi’an (China) [20], Baja California (Mexico) [21], Urayasu (Japan) [22], the Italian Peninsula [23], Istanbul (Turkey) [24], and Semarang (Indonesia) [25]. The second aspect is resolving two- or three-dimensional ground motion through the multi-sensor SAR images merging technique, which has been used in an urban subsiding area [26], mining subsiding area [27], and co-seismic deformation area [28]. The third aspect is developing the advanced algorithm to connect the multi-sensor SAR images so that the long-term time series deformation is observed [29]. However, there are no bibliographies available for ground deformation analysis with advanced multi-temporal InSAR for Kunming so far.

To better understand the spatiotemporal evolution of ground deformation in Kunming, a multi-temporal InSAR technique was utilized for the first time in this study to obtain a decade of time series deformation of Kunming. For this, 20 L-band Advanced Land Observation Satellite (ALOS-1) and 40 X-band Constellation of Small Satellites for Mediterranean basin Observation (COSMO-SkyMed) images were acquired in ascending and descending orbits, respectively. The annual deformation velocity was estimated to characterize the spatial pattern of subsidence in Kunming. Subsequently, comparisons were conducted to validate our results and the time series deformation was interpreted in detail. The causes of subsidence are discussed through correlation analysis.

## 2. Geological Setting of the Study Area

**Figure 2:**
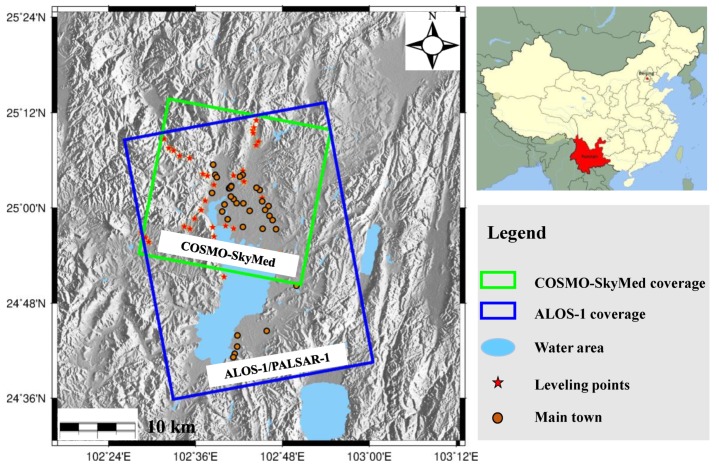
Study area and synthetic aperture radar (SAR) data coverage used in this study; corresponding data are superimposed on the digital elevation model (DEM).

The study area is located at Kunming, which is the capital of Yunnan province in southwest China, as shown in Figure 2. Situated in a fertile lake basin on the northern shore of Lake Dian and surrounded by mountains to the north, west, and east, Kunming is located at an altitude of 1900 m above sea level and at a latitude just north of the Tropic of Cancer [2]. As of 2014, Kunming has a population of 6.6 million, with an urban population of 4.5 million [30]. Due to the low latitude and high elevation, Kunming has one of the mildest climates in China, characterized by short, cool, dry winters with mild days and crisp nights, and long, warm, humid summers, which are still much cooler than the lowlands.

**Figure 3:**
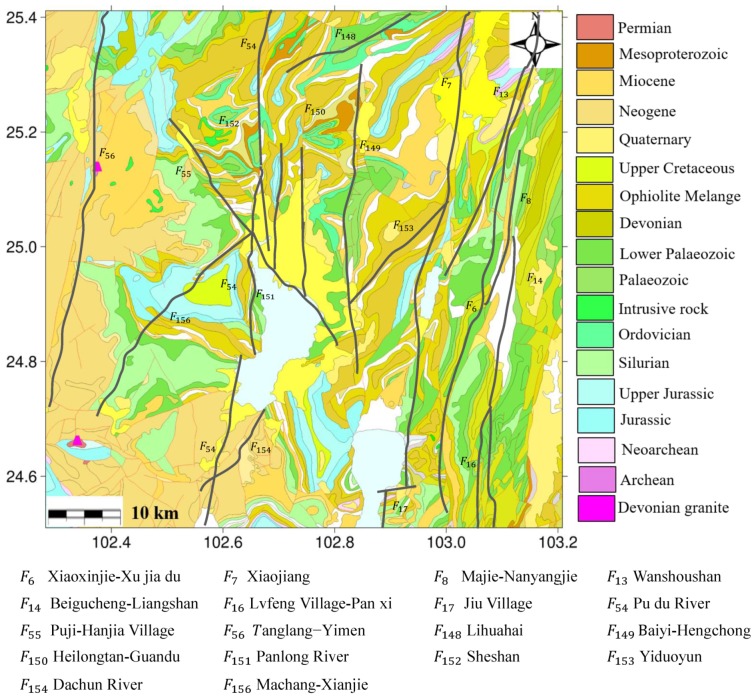
Geological map of study area. The black solid line represents quaternary active faults.

The study area is a late Cenozoic graben basin controlled by a number of quaternary active faults, e.g., the Heilongtan-Guandu fault (F_150_), Sheshan fault (F_152_), Pudu river fault (F_54_) and Puji-Hanjia village fault (F_55_) [4]. Figure 3 shows the geological map and location of faults in Kunming. The quaternary strata with different sediment thicknesses are widespread in the study area, including the lower, middle, and upper Pleistocene series as well as the Holocene series. Considering the composition, it is further divided into clay, muddy clay, and peaty soils [31]. These soft soils have many unfavorable properties regarding their use in projects, such as high compressibility, rheology, and thixotropy [1].

## 3. Materials and Methods

### 3.1. SAR Datasets

A total of 20 L-band ALOS-1 images with stripe mode and 40 X-band COSMO-SkyMed (CSK) images were collected to derive the time series deformation over Kunming. Table 1 and Figure 2 show the parameters and coverages of these SAR images, respectively. The ALOS-1 images with an ascending orbit and stripe mode were acquired between January 2007 and March 2011. To maintain the temporal continuity, CSK images with ascending orbit were acquired between June 2011 and January 2016. Based on these two types of SAR data, we were able to obtain the ground deformation over the study area between January 2007 and January 2016. In order to validate the obtained deformation, additional SAR images and leveling data were employed in this study, including 91 Sentinel-1A images and 37 leveling points (represented by red stars in Figure 2). A light detection and ranging (LIDAR) digital elevation model (DEM), which had a spatial resolution of 3 m and centimeter-level height precision, was acquired as an external DEM to remove the topographic phase from the differential interferograms [4].

### 3.2. Multi-Temporal InSAR Processing

Using the collected 20 L-band ALOS-1 and 40 X-band CSK images, time series of deformation was derived from the multi-temporal InSAR technique with GAMMA Remote Sensing and Consulting AG (GAMMA) SAR software. This process included three steps.

**Figure 4:**
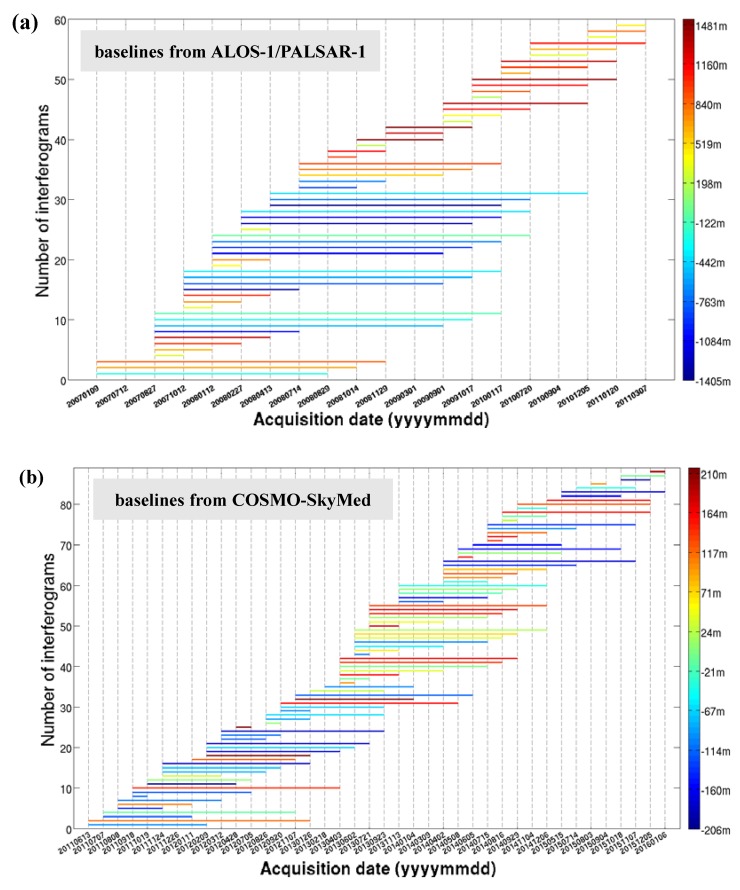
The distribution of the temporal and spatial baselines from (**a**) Advanced Land Observation Satellite (ALOS-1) and (**b**) Constellation of Small Satellites for Mediterranean basin Observation (COSMO-SkyMed) in this study.

Firstly, interferometric pairs were generated by setting small temporal and spatial baselines. This depressed noise effects and maintained a high level of coherence [32]. On the basis of the experiment, a spatial baseline below 1500 m and a temporal baseline less than 1000 days were designed for the L-band SAR images to generate the interferograms. For the X-band SAR images, a spatial baseline below 220 m and a temporal baseline less than 200 days were designed to generate the interferograms. A total of 59 interferograms from the L-band (Figure 4a) and 88 interferograms from the X-band (Figure 4b) were produced for the time series deformation estimation.

Secondly, after selection of the point targets, removal of the topographic and orbital phases, filtering of the interferograms, and retrieval of the absolute phase, unwrapped interferograms were produced. The process of point target selection was based on the method in [33]. The topographic and orbital phases were simulated and then removed from the interferometric phase using collected LIDAR DEM data and SAR orbital data. Then, the process of adaptive spectral filtering with a window size of 64 for the L-band and 32 for the X-band was executed to suppress the interferometric noise [34]. Finally, phase unwrapping using the minimum cost flow (MCF) method was carried out to retrieve the absolute phase [35]. During this procedure, a common point target was selected as the reference point to maintain consistency of results from the L- and C-bands.

Thirdly, the time series deformation was estimated from the collected L- and X-band images. After the second step, the unwrapped interferometric phase may consist of residual phase errors, such as topographic phase error, orbital error, or atmospheric delay error. Prior to the time series deformation estimation, these residual phase errors should be removed from the unwrapped interferometric phase. In this case, the topographic phase error was safely neglected since high-precision LIDAR DEM was used to simulate the topographic phase. A quadratic polynomial phase model was fitted to reduce the orbital phase error [36] and the residual atmospheric delay error, including atmospheric and ionospheric delay, was mitigated by spatial low-pass filtering and temporal high-pass filtering [33]. After these processes, the residual phase errors were finally reduced and the high-precision time series deformation was produced.

## 4. Results and Analysis

### 4.1. Annual Deformation Velocity

**Figure 5:**
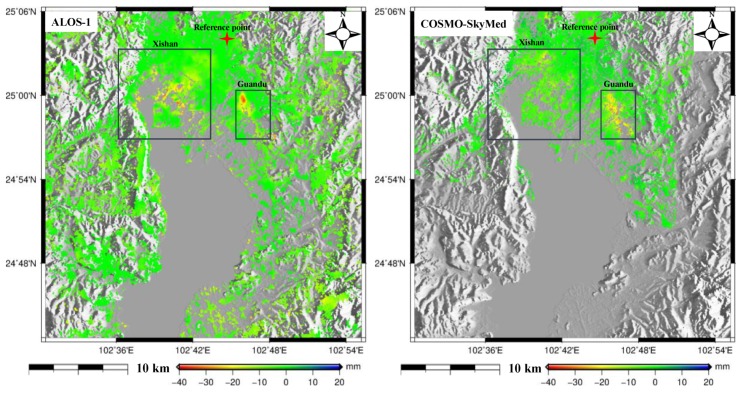
The annual deformation velocity maps from ALOS-1 (**left**) and COSMO-SkyMed (**right**) in this study. The red cross shows the reference point and the black rectangles denote the subsiding region.

The annual deformation velocity maps in the vertical direction were derived from 59 L-band interferograms and 88 X-band interferograms, as shown in Figure 5; the left figure shows the result from the L-band ALOS-1 and the right figure shows the result from the X-band CSK. It should be noted that we assumed that the deformation of Kunming was dominated by the vertical direction and was insignificant in the horizontal direction when converting the line-of-slight (LOS) deformation into the vertical deformation. Actually, it was acceptable for our region as the deformation was mostly vertical [5,6,7]. The statistics in Figure 5 show that a large portion of the deformation measurements was strongly skewed toward negative values, indicating that most areas of Kunming are in a state of subsidence. High subsidence rates were observed to be concentrated in two regions (Xishan and Guandu towns, which are marked with black rectangles in Figure 5), where the maximum subsidence reached 35 mm/y using ALOS-1 (left of Figure 5) and 30 mm/y using X-band COSMO-SkyMed (right of Figure 5).

**Figure 6:**
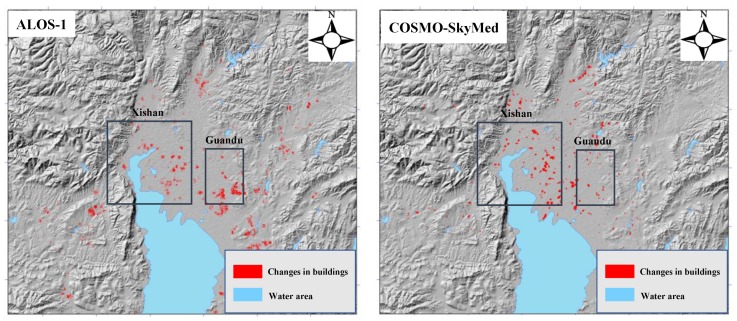
Changes in buildings from ALOS-1 (**left**) and COSMO-SkyMed (**right**) in this study.

Careful inspection of Figure 5 suggests that there was a subtle difference in the subsiding magnitude between the L- and X-band results; the L-band (left) illustrated a larger subsidence than the X-band. This phenomenon was particularly obvious for the marked region in Figure 5. This difference may have been caused in two ways. The first reason involves the different SAR-acquired times, i.e., the L-band result was derived between January 2007 and March 2011 while the X-band result was derived between June 2011 and January 2016. Clearly, there was no overlapping time between the L- and X-bands. As shown in the introduction, the subsidence in Kunming was highly correlated with urbanization and groundwater extraction, meaning that the degree of urbanization and groundwater extraction with respect to the different times may have led to the different deformation magnitudes [5]. Based on this analysis, the relevant information was investigated and the degree of urbanization and groundwater extraction was found to be extremely intensive in Kunming before 2010 [4]. After this date, Kunming was controlled by the local government, resulting in the slowing down of urbanization and the rise of the groundwater level. In order to further display this investigation, changes in buildings (an index to reflect the degree of urbanization) were extracted through analysis of SAR amplitude and coherence images [37]. Here, changes in buildings mainly refer to new buildings, which were easily identifiable from SAR amplitude and coherence images. The left and right panels of Figure 6 show the changes in buildings from January 2007 to March 2011 and June 2011 to January 2016, respectively. The statistics in Figure 6 indicate that the area of new buildings was larger in the left panel than the right, demonstrating more intensive urbanization during the ALOS-1 SAR-acquired time. From these investigations, the L-band showed more subsidence than the X-band due to more intensive urbanization and groundwater extraction. The second reason why different subsidence magnitudes were apparent between the L- and X-bands lies in the different imaging geometry, i.e., the L-band, with an incidence angle of 38°, belongs to the ascending orbit, while the X-band, with incidence angle of 29°, belongs to the descending orbit. According to the relationship between LOS deformation and three-dimensional (north, east, and up) deformation, the three-dimensional vectors of LOS direction are [– 0.1069 0.6063 0.7880] for the L-band and [0.0842 0.4774 0.8746] for the X-band [38]. As indicated by previous studies, the deformation of Kunming was dominated by the vertical direction and was insignificant in the horizontal direction (north and east) [5,6,7]. Therefore, the northern and eastern components were directly ignored when converting the LOS deformation into the vertical deformation. In this research, the conversion factor for the L-band was larger than the X-band when connecting the different three-dimensional vectors, which may have partly caused the larger subsidence in the L-band than the X-band.

### 4.2. Validation of InSAR Results

#### 4.2.1. Comparison with Leveling-Derived Deformation

**Figure 7:**
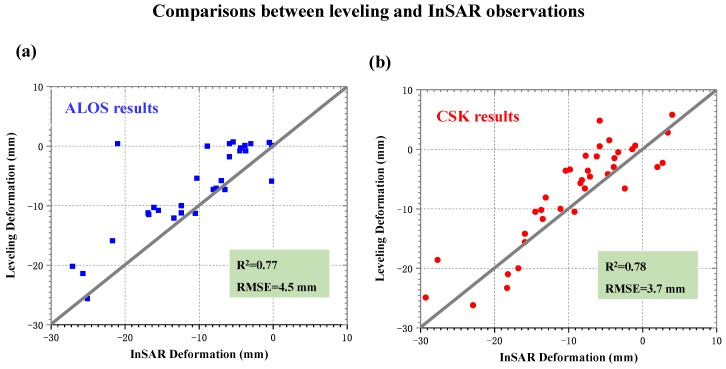
Comparison of annual deformation velocity between leveling and InSAR from (**a**) the L-band ALOS-1 and (**b**) the X-band COSMO-SkyMed.

In order to validate our results, the annual deformation velocities between leveling and InSAR were compared, as shown in Figure 7, where the left and right figures show the results from the L-band ALOS-1 and the X-band CSK, respectively. The leveling-measured deformation velocity was mainly derived from the local earthquake-monitoring department, so most of these instances were not located in severely subsiding areas and therefore show relatively little deformation [4]. To ensure the comparability of our results, the mean value within a 50 m radius circle with a leveling point center (as shown by the red stars in Figure 2) was extracted from Figure 5 using the InSAR observations. The statistics in Figure 7 show that the root mean square error (RMSE) values between InSAR and the leveling observations were about 4.5 mm for the L-band and 3.7 mm for the X-band. The higher accuracy for the X-band may have been due to more interferograms being involved in the annual deformation velocity estimation for the X-band (88 interferograms) than L-band (59 interferograms). This comparison is approximately consistent between InSAR and the leveling observations, demonstrating the reliability of our observations in Kunming.

#### 4.2.2. Comparison of Deformation Between the ALOS-1 and Sentinel-1A Datasets

**Figure 8:**
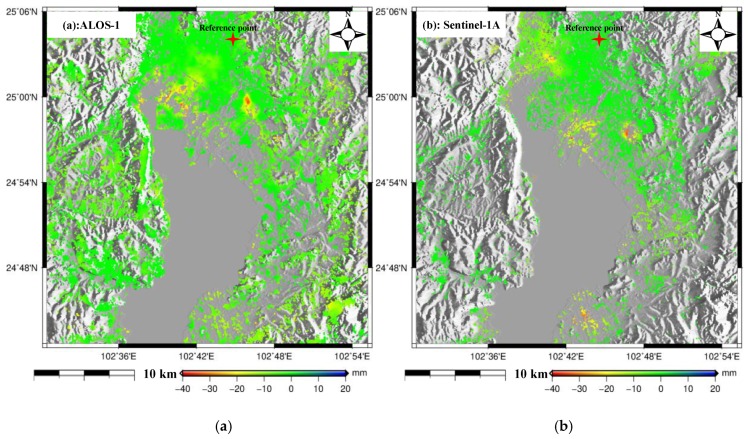
Comparison of deformation between the ALOS-1 and Sentinel-1A datasets. (**a**) The result of ALOS-1. (**b**) The result of Sentinel-1A.

**Figure A1:**
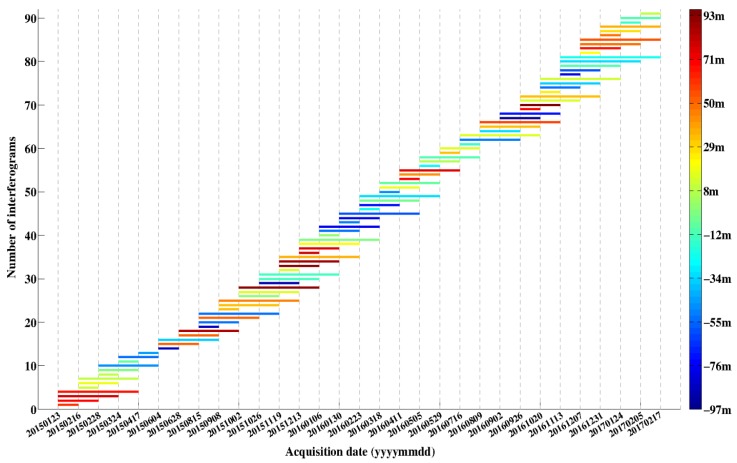
The distribution of the temporal and spatial baselines from Sentinel-1A with ascending orbit between 23 January 2015 and 17 February 2017.

**Figure A2:**
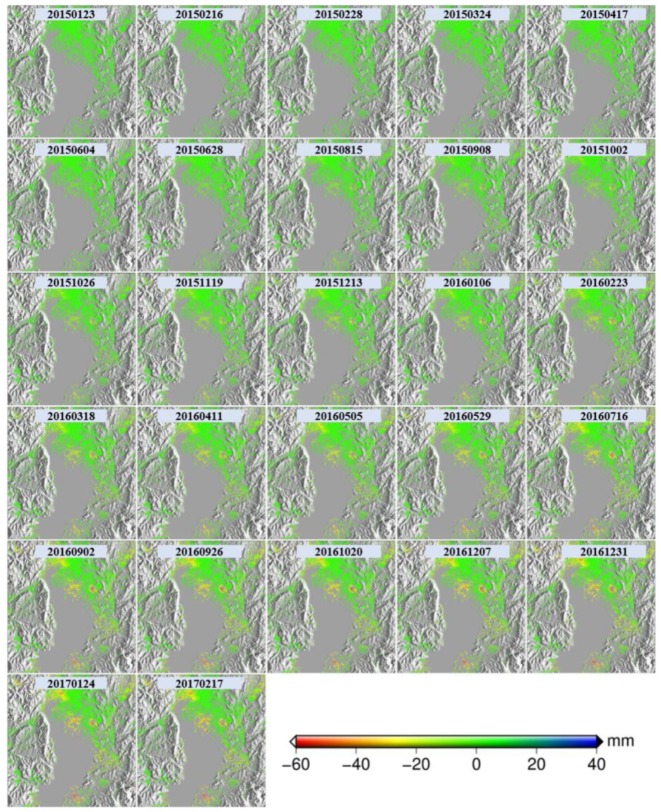
Time series deformation in Kunming observed using C-band Sentinel-1A images with ascending orbit between 23 January 2015 and 17 February 2017.

Thirty-one additional Sentinel-1A images with ascending orbit (Table 1) were processed using the multi-temporal InSAR technique to generate the ground deformation between 23 January 2015 and 17 February 2017 with the purpose of comparing with ALOS-derived deformation. After setting the spatial-temporal baselines (Figure A1 in the Appendix A), the time series deformation was produced over the study area, as shown in Figure A2 in the Appendix A. Due to no overlapping time between these two datasets, the annual deformation velocity maps were compared to investigate the relationship, as shown in Figure 8a,b; Figure 8a shows the result from L-band ALOS-1 and Figure 8b shows the result from C-band Sentinel-1A. It was observed that there was clear difference in the distribution of subsiding regions between these two results; both Guandu and Xishan subsiding regions (black rectangles in Figure 5) in Figure 8a moved about five kilometers to the southeast in Figure 8b; the obvious subsidence appeared in the upper left and bottom of Figure 8b, while this was not significant in Figure 8a. We think this inconsistency is due to the different SAR-acquired times, i.e., the ALOS-1 result was derived between January 2007 and March 2011 while the Sentinel-1A result was derived between January 2015 and February 2017. This comparison also indicates that the distribution of ground subsidence in Kunming varied dynamically with time. It needs to be emphasized that there are not suitable SAR images with ascending orbit during our ALOS-acquired period. Therefore, it is difficult to validate the ALOS-derived ground deformation by comparing with additional SAR datasets. However, we think the ALOS result was reliable when comparing with the leveling-derived deformation.

#### 4.2.3. Comparison of Deformation Between the COSMO-SkyMed and Sentinel-1A Datasets

**Figure 9:**
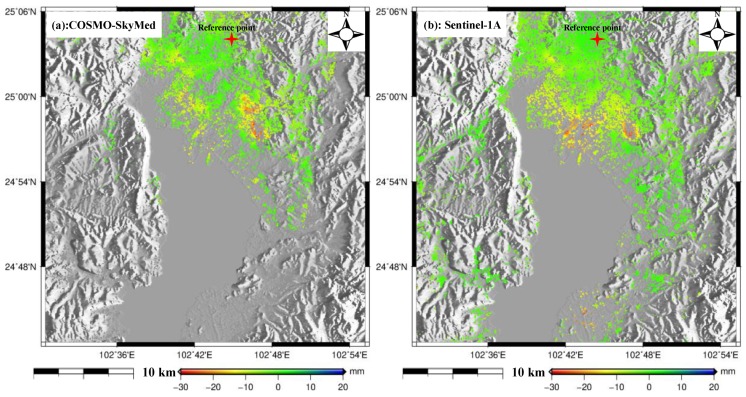
Comparison of deformation between the COSMO-SkyMed and Sentinel-1A datasets. The Figure 9a show the result of COSMO-SkyMed and Figure 9b show the result of Sentinel-1A.

**Figure A3:**
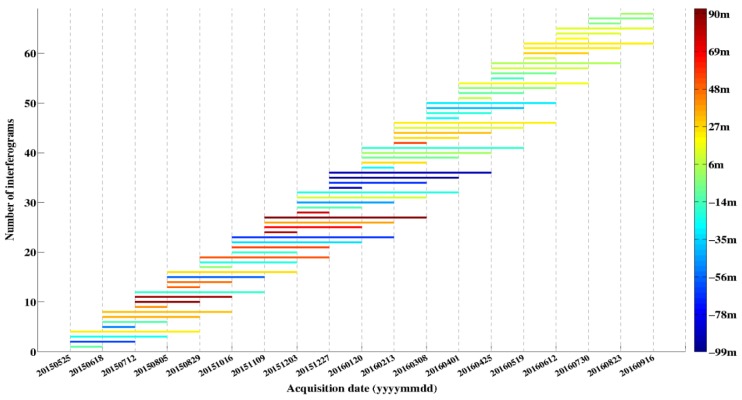
The distribution of the temporal and spatial baselines from Sentinel-1A with descending orbit between 25 May 2015 and 16 September 2016.

**Figure A4:**
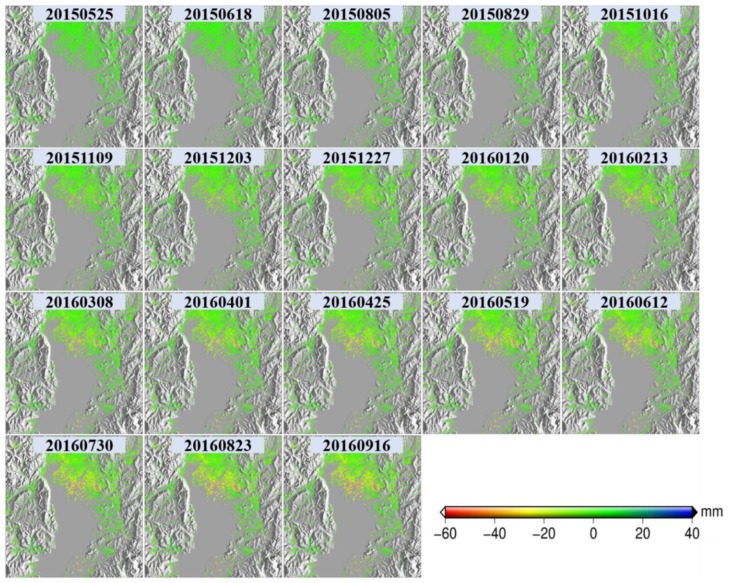
Time series deformation in Kunming observed using C-band Sentinel-1A images with descending orbit between 25 May 2015 and 16 September 2016.

**Figure A5:**
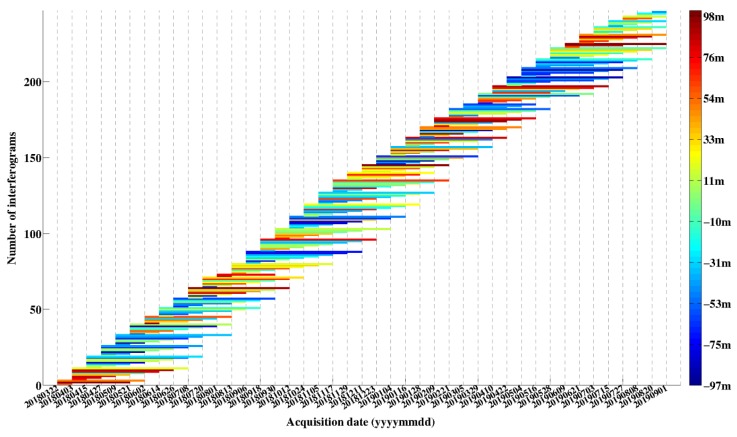
The distribution of the temporal and spatial baselines from Sentinel-1A with descending orbit between 22 March 2018 and 1 September 2019.

**Figure A6:**
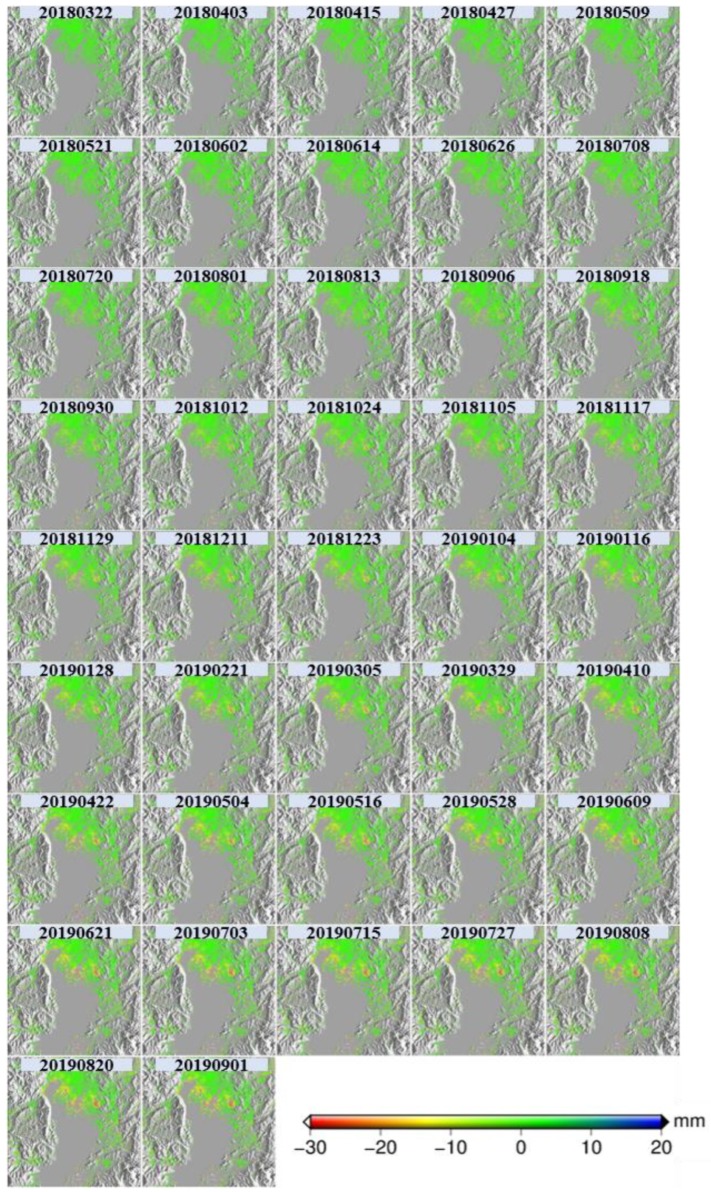
Time series deformation in Kunming observed using C-band Sentinel-1A images with descending orbit between 22 March 2018 and 1 September 2019.

To further validate the CSK-derived deformation, 60 C-band Sentinel-1A images (Table 1) with descending orbit were processed using the multi-temporal InSAR technique to generate the time series deformation. This procedure was divided into two phases due to lack of data between October 2016 and March 2018. The first phase generated the time series deformation from 25 May 2015 to 16 September 2016 through processing 18 C-band Sentinel-1A images, as shown in Figure A3 and Figure A4 in the Appendix A. The second phase generated the time series deformation from 22 March 2018 to 1 September 2019 through processing 42 C-band Sentinel-1A images, as shown in Figure A5 and Figure A6 in the Appendix A. It was observed from Figure A4 and Figure A6 that the Guandu and Xishan subsiding regions in Figure 5 moved about five kilometers to the southeast, which was consistent with Figure A2. To ensure the consistency of time, the CSK-derived deformation map spanning from 15 May 2015 to 6 January 2016 (Figure 9a) was compared with Sentinel-derived deformation spanning from 25 May 2015 to 20 January 2016 (Figure 9b). Figure 9a was resampled to the geographical coordinate system as in Figure 9b. Unlike the last comparison, the distribution of subsiding region was basically consistent between Figure 9a,b. However, the slight difference was that the CSK showed a larger subsidence than Sentinel-1A in the north of Guandu subsiding region. This phenomenon may have been caused by the different wavelengths; CSK belongs to the X-band while Sentinel-1A belongs to the C-band.

### 4.3. Time Series Deformation in Guandu

**Figure 10:**
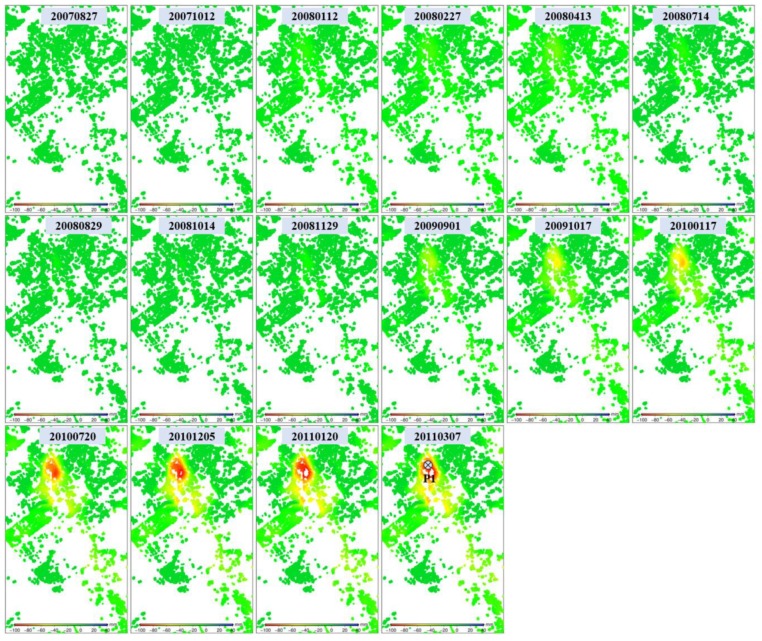
Time series deformation in Guandu (marked with black rectangles in Figure 5) observed from L-band ALOS-1 images. The time series deformation at point P1 will be extracted for further analysis.

As shown in Figure 5, the regions Guandu and Xishan, which both suffer from severe subsidence, were observed with the L- and X-band SAR interferograms. To obtain detailed information relating to these two regions, the time series deformation was estimated using multi-temporal InSAR processing, as described in Section 3.2. Figure 10 shows the time series deformation of Guandu from 27 August 2007 to 7 March 2011, which was derived from L-band ALOS-1 images, and also shows prominent subsidence in the upper center of the figure, where the cumulative maximum subsidence reached 110 mm. It was also found that the subsidence presented in the shape of a funnel and extended to the southeast. The decorrelation in the southeast was due to ongoing construction [4], as shown in Figure 6.

**Figure 11:**
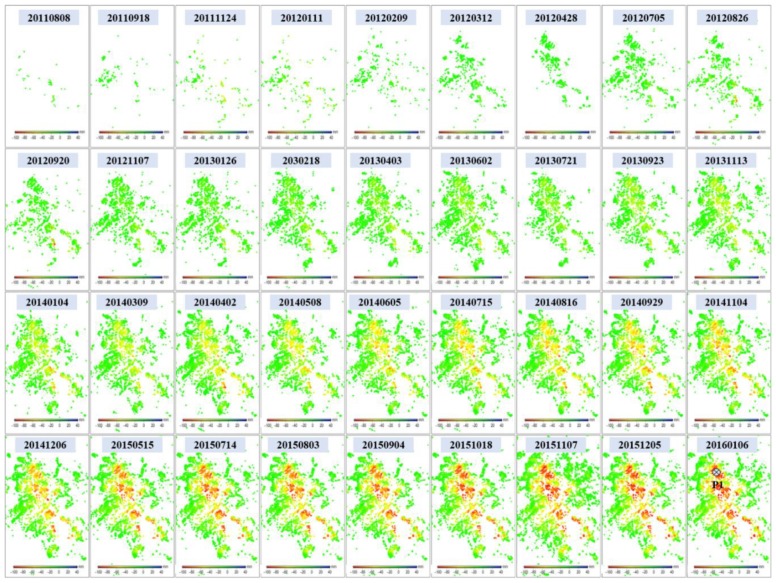
Time series deformation in Guandu (marked with black rectangles in Figure 5) observed using X-band COSMO-SkyMed images. The time series deformation at point P1 will be extracted for further analysis.

Figure 11 shows the time series deformation of Guandu between 8 August 2011 and 6 January 2016, which was derived from the X-band CSK images. Similarly to Figure 10, prominent subsidence started from the upper center and extended to the southeast. However, most of the subsiding region in the southeast kept high coherence, as shown in Figure 11, while it was decorrelated in Figure 10. This phenomenon can be explained by the fact that most construction during the L-band SAR-acquired time was completed during the X-band SAR-acquired time. The statistics indicate that the cumulative maximum subsidence was 102 mm, which was basically consistent with the L-band result.

**Figure 12:**
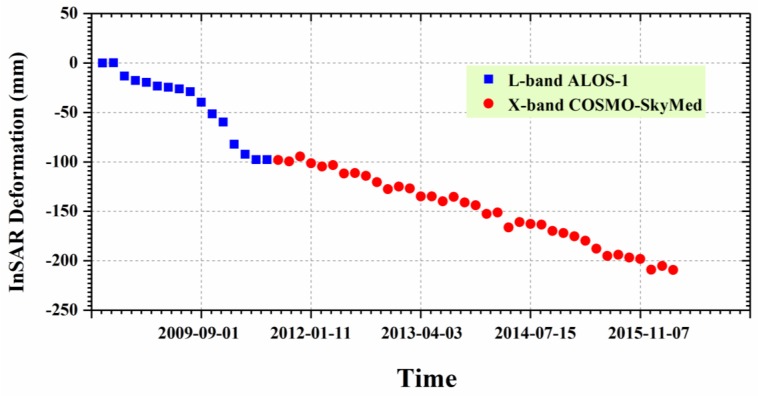
Time series deformation at point P1 marked in Figure 10 and Figure 11. The blue squares and red circles represent deformation for the L-band and the X-band, respectively.

To investigate the long-term ground deformation time series, both L-band ALOS-1 and X-band CSK deformation in LOS direction were projected to the vertical direction. After that, the cumulative vertical deformation from the L-band was added to the X-band to connect them. Figure 12 shows the vertical time series deformation of Guandu at point P1 between 27 August 2007 and 6 January 2016. The blue squares and the red circles represent the vertical deformation values for the L-band and the X-band, respectively. It was observed that the deformation at point P1 presented an approximately linear variation with time, particularly for the X-band. The cumulative subsidence reached 209 mm during the period of 2007–2016.

### 4.4. Time Series Deformation in Xishan

**Figure 13:**
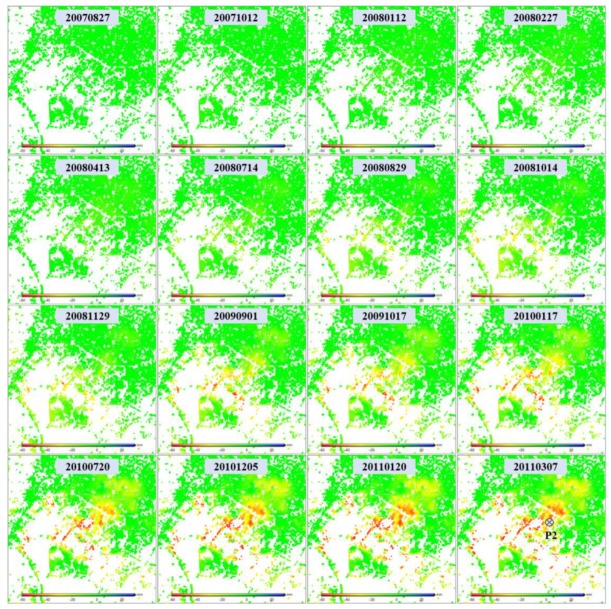
Time series deformation in Xishan (marked with black rectangles in Figure 5) observed using L-band ALOS-1 images. The time series deformation at point P2 will be extracted for further analysis.

Like Guandu, Xishan is another region with severe subsidence. Figure 13 displays the time series deformation of Xishan from 27 August 2007 to 7 March 2011 derived from L-band ALOS-1 images. Compared with Guandu, subsidence was not significant in Xishan, with the cumulative deformation ranging from − 60 mm to 10 mm during the ALOS-1 SAR-acquired time. The subsidence presented in a long rectangle shape and extended southwest in Xishan, which was different to Guandu. Further observation indicated that the subsiding points in Xishan were not as concentrated as in Guandu, showing a certain discreteness. This phenomenon was related to the distribution of high-rise buildings [29].

**Figure 14:**
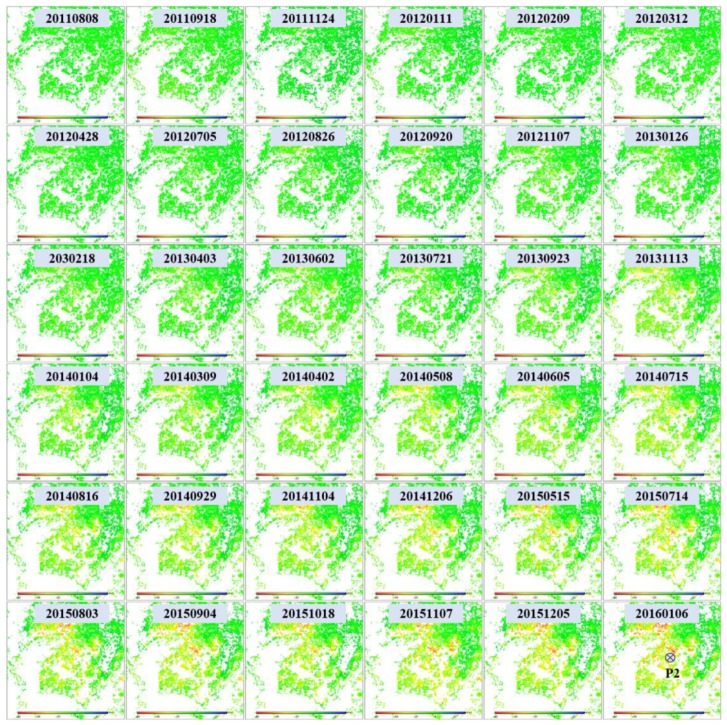
Time series deformation in Xishan (marked with black rectangles in Figure 5) observed from X-band COSMO-SkyMed images. The time series deformation at point P2 will be extracted for further analysis.

The time series deformation of Xishan from 8 August 2011 to 6 January 2016 was observed from the X-band CSK images, as shown in Figure 14. It was found that the point targets in Figure 14 were denser than those in Figure 13, which may have benefited from the higher spatial resolution of the X-band than the L-band [18]. Similar to Figure 13, the subsidence was approximately in the shape of a long rectangle that extended to the southwest. However, the obvious subsidence appeared in the upper left in Figure 14, while this was not significant in Figure 13. This difference was due to the new completed constructions, which was confirmed by filed investigation [4]. The statistics show that the cumulative maximum subsidence was 52 mm during this period, which was slightly less than the L-band result.

**Figure 15:**
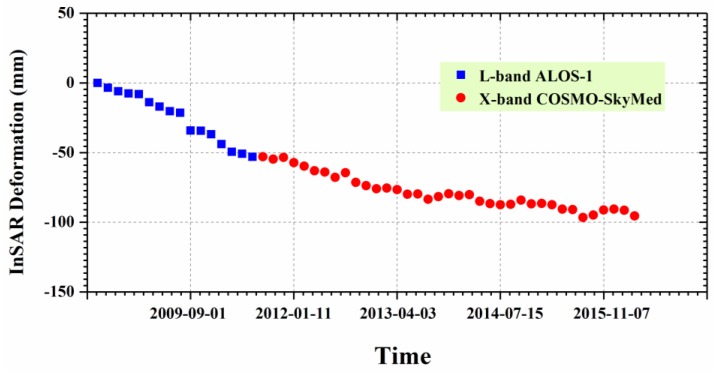
Time series deformation at point P2 marked in Figure 13 and Figure 14.

Figure 15 shows the vertical time series deformation of Xishan at point P2 from 27 August 2007 to 6 January 2016, where the blue squares and red circles represent the vertical deformation for the L-band and the X-band, respectively. This was similar to that observed in Figure 12, in that the cumulative deformation from the L-band was added to the X-band result after converting the LOS deformation into the vertical deformation. Figure 15 shows that the deformation at point P2 presented an approximately linear variation with time, where the cumulative subsidence was 98 mm during the period of 2007–2016.

## 5. Discussion

### 5.1. Subsidence Due to Soft Soil Consolidation

**Figure 16:**
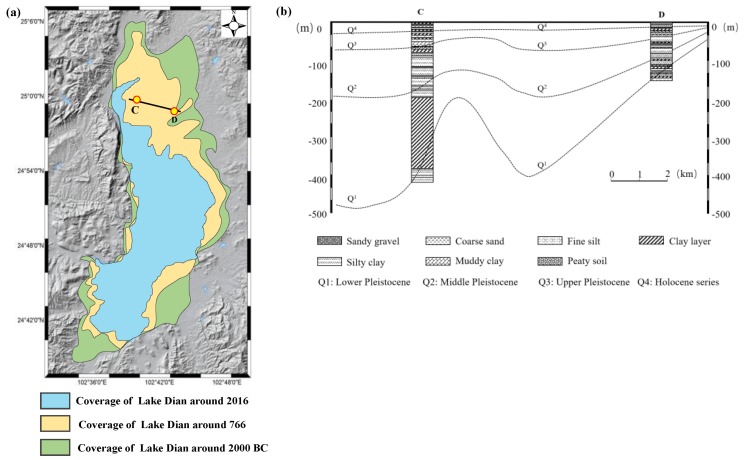
(**a**) Changes in the shoreline of Lake Dian; (**b**) stratigraphic profile along the line of CD, which corresponds to the position of boreholes.

Kunming was a typical lacustrine sediment basin that was filled by a large area of soft soil [1]. Figure 16a, which displays the changes in the shoreline of Lake Dian, clearly indicates the development of Kunming basin. In this context, the stratum of Kunming was mainly composed of clay, muddy clay, and peaty soils. These soft soils had many unfavorable properties regarding their use in projects, such as high compressibility, rheology, and thixotropy [4]. Subsidence easily occurred when soils had high compressibility due to the natural consolidation of the soils and man-made building load [39]. Figure 16b shows the stratigraphic profile along the line of CD (black solid line in Figure 16a), which corresponds to the position of boreholes. It was found that both Xishan and Guandu were filled with different layers of soft soils with different quaternary thicknesses. Based on such a stratigraphic structure, it was deduced that soft soil consolidation was one of the factors causing the severe subsidence observed in the study area.

**Figure 17:**
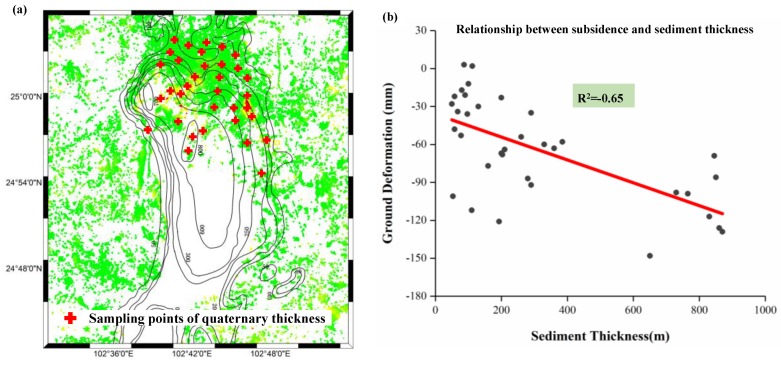
(**a**) Quaternary sediment thickness contours in Kunming and (**b**) relationship between ground deformation and quaternary sediment thickness.

The relevant materials show that the magnitude of subsidence may be related to the quaternary sediment thickness [40]. Thus, the relationship between ground deformation and quaternary sediment thickness was investigated and analyzed over the study area. The quaternary sediment thickness of Kunming was collected from the local geological department, then the spatial distributions of the deformation and the quaternary sediment thickness were compared, as shown in Figure 17a. The comparison results show the approximate consistency between the ground deformation and the quaternary sediment thickness in the spatial distribution; the greater the subsidence, the thicker the quaternary, particularly for the subsiding region of Xishan, where the sediment thickness was 500–800 m [4]. To further investigate this correlation, regression analysis was carried out for ground deformation and quaternary sediment thickness at the selected sampling points. In order to ensure the rationality of comparison, points with different quaternary sediment thickness were randomly selected in this study, as shown in Figure 17a. After collecting the information of quaternary sediment thickness, the mean value within a 50 m radius circle with a sampling point center was extracted from L-band ALOS-derived deformation as the InSAR observations, as shown in Figure 17b [40]. It was found that some large subsidence points corresponded to the thick quaternary sediment, while some small subsidence points corresponded to the shallow quaternary sediment. The statistics show that the correlation coefficient between these two factors is about −0.65, suggesting that there is somewhat negative correlation between ground deformation and quaternary sediment thickness. When ignoring the other factors (e.g., building load, construction), our result indicates some degree of correlation between the ground deformation and soft soil consolidation. However, it was also observed that there were some inconsistent points, e.g., the large subsidence but shallow sediment seen in Figure 17b. This inconsistency may be due to the heterogeneous thickness of the compressible soils across the quaternary deposits or due to the other factors causing the severe subsidence observed in the study area [40]. Based on the analyses of the stratigraphic structure in Kunming (Figure 16) and the relationship between ground deformation and quaternary sediment thickness (Figure 17), it was deduced that unconsolidated sedimentary deposits was one of the factors causing the severe subsidence in Kunming.

**Figure 18:**
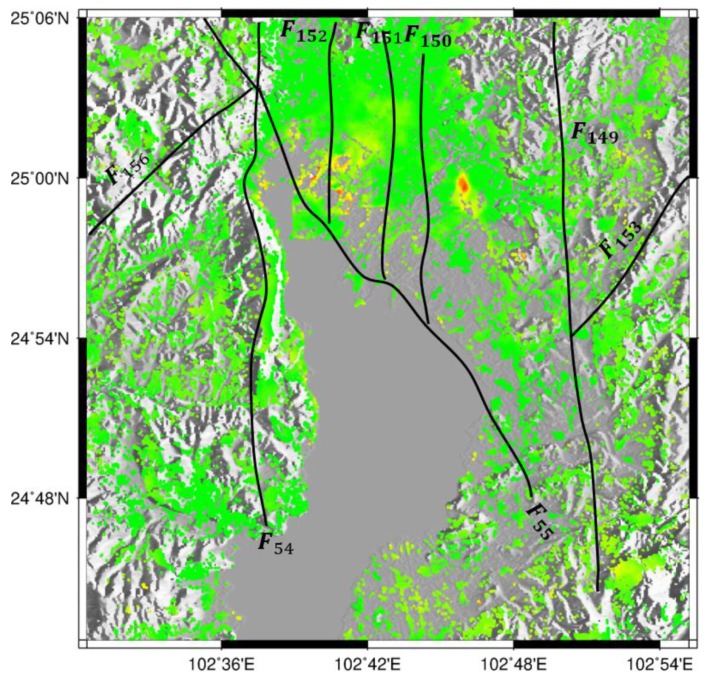
The relationship between ground deformation and quaternary active faults in Kunming. The black solid line shows the quaternary active faults.

For further investigation of the relationship between ground deformation and geological setting, the quaternary active faults were superimposed on the L-band ALOS-derived deformation map, as shown in Figure 18, where the black solid line represents the quaternary active faults. It was observed that there were eight faults around Kunming, which controlled the formation and development of the basin. As many studies have indicated, the spatial distribution and shape of subsidence might be related with the faults [19,20,21]. In this study, Guandu subsiding region presented in the shape of a narrow funnel and extended in an approximate south-north direction, which followed the general trends of the surrounding F_149_ and F_150_ faults. The Xishan subsiding region presented in a long rectangle shape and extended to the southwest, which didn’t follow the general trends of the surrounding F_151_, F_152_, and F_55_ faults. However, careful inspection suggests that Xishan subsiding region might be bounded by these three faults: the east was bounded by F_152_, the west and south were bounded by F_55,_ and F_152_ acted as barrier to impede the horizontal propagation of deformation on both sides. Based on this analysis, there was a certain degree of correlation between ground deformation and quaternary active faults over the area.

### 5.2. Subsidence Due to High-Rise Building Load

**Figure 19:**
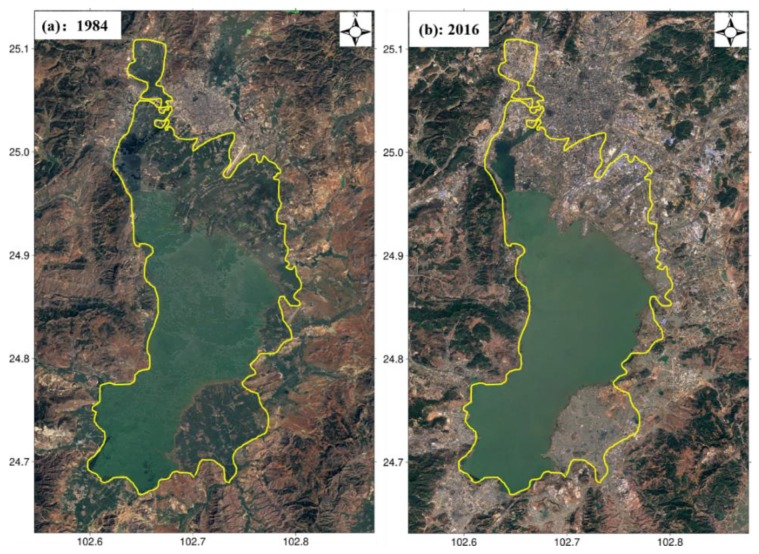
Changes in land cover over Kunming from (**a**) 1984 to (**b**) 2016; the yellow curve shows the main changes.

**Figure 20:**
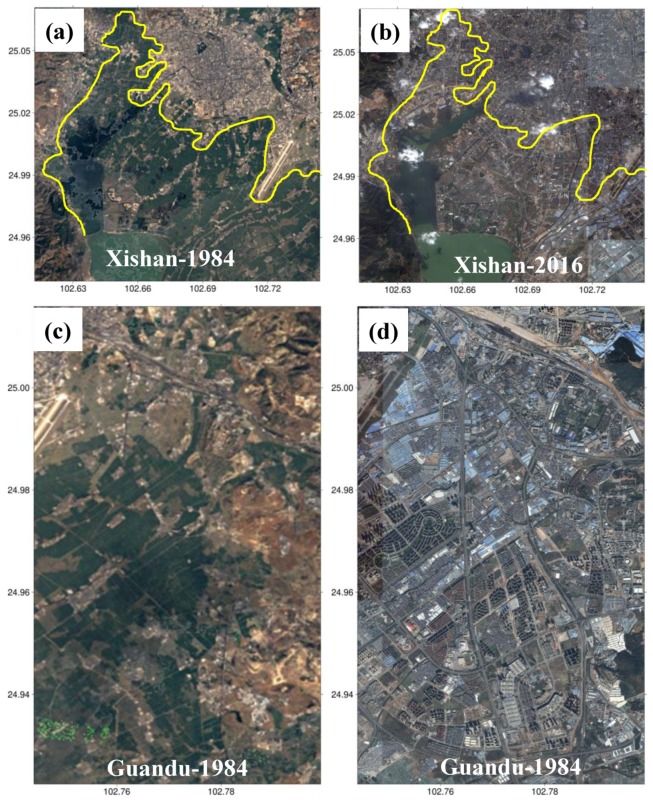
The changes in land cover over Xishan from (**a**) 1984 to (**b**) 2016, and over Guandu from (**c**) 1984 to (**d**) 2016.

As introduced in Section 2, Kunming is surround by mountains to the north, west, and east, and by a lake to the south. Under these condition, available space is very limited and rapid urbanization in Kunming is inevitable. To solve this problem, a large number of high-rise buildings with high building density were set up around Lake Dian over the last few decades. Figure 19 shows the changes in land cover from 1984 to 2016, where the yellow curve indicates the main range of changes. It is clear that a large area of green land was occupied by high-rise buildings and other structures. This change was more obvious in Xishan and Guandu, which corresponded to the severely subsiding regions, as shown in Figure 20. Considering the soft soil foundations in Figure 16, this high-rise building load inevitably caused the severe subsidence in Kunming, particularly for Xishan and Guandu [41,42].

**Figure 21:**
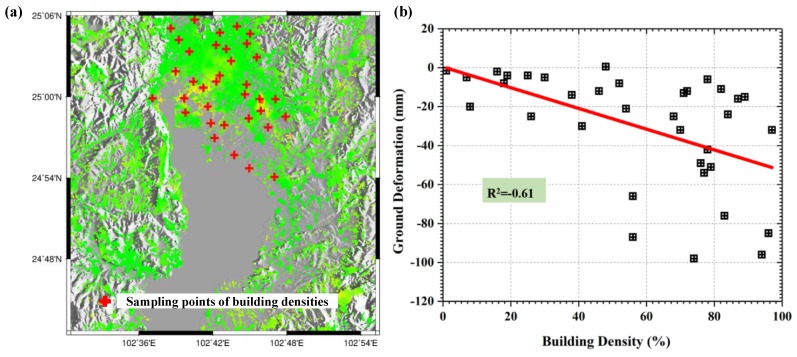
The distribution of sampling points of building densities (**a**) and the relationship between ground deformation and building density (**b**).

To further investigate the relationship between subsidence and building load, the building densities over 35 sampling points were extracted and analyzed over Kunming. Building density is the floor area of the building divided by the total area of the site [43]. It should be noted here that the floor area of a building only refers to the single floor area and doesn’t contain the height information. The information of 35 sampling points was collected through filed investigation organized in June, 2016. In order to ensure the rationality of comparison, buildings with different densities were investigated and analyzed in this study. The selected sampling points were mainly concentrated in the regions sensitive to ground deformation, such as residential areas with high-rise buildings, government administrative areas, dense business districts, as well as important infrastructure regions, as shown in Figure 21a. After collecting the information of building densities, the mean value within the investigated area was extracted from L-band ALOS-derived deformation as the InSAR observations, as shown in Figure 21b. The statistics show that the correlation coefficient between ground deformation and building density was about −0.61, suggesting somewhat negative correlation between them. When ignoring the other indexes (e.g., building height) and assuming that building density was the only index to reflect the building load, our result indicates some degree of correlation between the ground deformation and building load. However, it was also observed that there were some inconsistent points, e.g., the large subsidence but small density seen in Figure 20. This inconsistency may be due to the other factors causing the severe subsidence. Based on the analyses of land cover change in Kunming (Figure 19 and Figure 20) and the relationship between ground deformation and building density (Figure 21), it was deduced that building load was one of the factors causing the severe subsidence in Kunming, particularly for Xishan and Guandu.

### 5.3. Subsidence Due to Groundwater Exploitation

**Figure 22:**
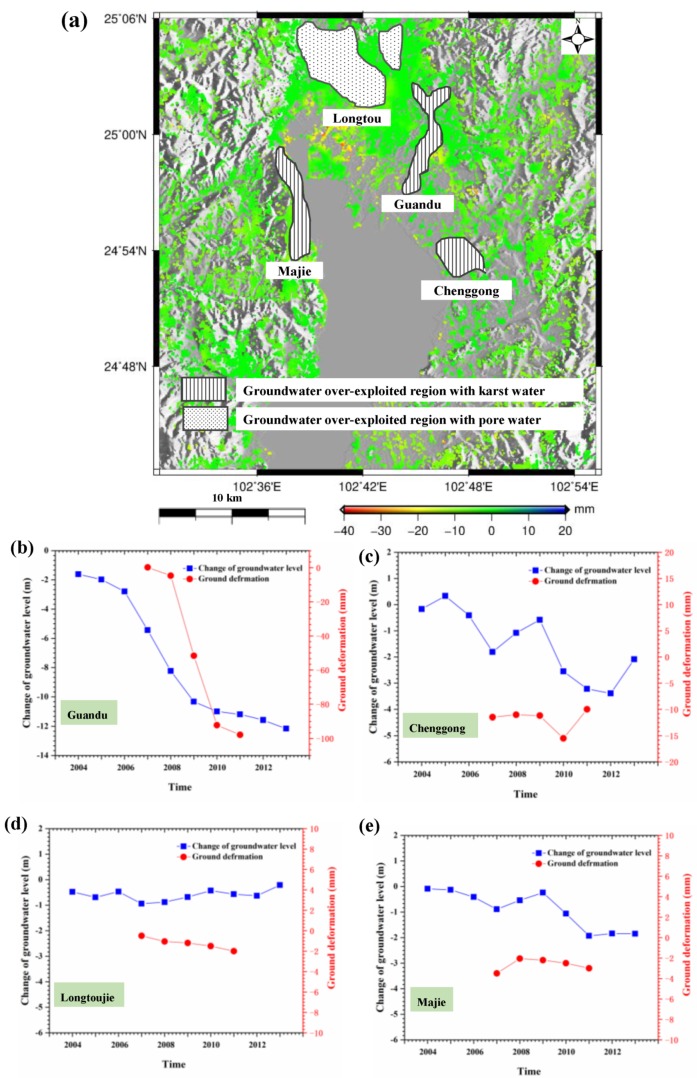
The distribution of groundwater exploited regions in Kunming (**a**) and relationship between annual changes of groundwater level and ground deformation in Guandu (**b**), Chenggong (**c**), Longtoujie (**d**), and Majie (**e**). The ground deformation is derived from L-band ALOS-1.

Groundwater is the main water supply in Kunming, where the average daily groundwater exploitation was more than 170,000 m^3^ prior to 2010 [4]. Figure 22a shows the distribution of groundwater exploited regions in Kunming, which corresponds to the groundwater supplying sources. It was found that exploited regions mainly lie in eastern Guandu and Chenggong, northern Longtoujie, and western Majie. Among them, Guandu, Chenggong, and Majie belong to the karst groundwater, and Longtoujie belongs to the pore groundwater. According to the observations of groundwater levels, eastern Guandu declined 12.1 m between 2004 and 2012, which was the greatest decline in Kunming, as shown in the blue line of Figure 22b [44]. It was found that there was a dramatic decline between 2006 and 2010 and, subsequently, a slight decline between 2010 and 2012. This variation was consistent with the time that restrictions were placed on groundwater extraction. As many pioneer studies indicated, groundwater exploitation certainly resulted in ground subsidence [45,46]. Therefore, severe subsidence was observed in Guandu, as shown in the red line of Figure 22b, where the cumulative subsidence reached 100 mm during the period of 2007–2011. Comparison between the change of groundwater level and ground deformation in Figure 22 indicates that they presented similar variations in temporal domain. For the other exploited regions, the changes of groundwater level weren’t as significant as Guandu, and ground deformation was also not as severe, as shown in Figure 22c–e. Based on the analyses of groundwater exploited regions and level changes, it was deduced that groundwater exploitation was one of factors causing the severe subsidence in Kunming, particularly for Guandu.

## 6. Conclusions

With the development of urbanization, ground subsidence has become one of the most prominent geological hazards in Kunming and has caused substantial damage to homes, roads, canals, pipelines, and other types of infrastructure. In this study, a decade of time series deformation in Kunming was derived using a multi-temporal InSAR technique to provide scientific evidence regarding the sound management of urbanization. Based on this study, the following conclusions were made.

(1) Two severe subsiding regions were observed using L- and X-band SAR data. Using 20 L-band ALOS-1 images and 40 X-band COSMO-SkyMed images, the annual deformation velocity and time series deformation maps of Kunming were retrieved through multi-temporal InSAR processing. The results showed that most areas of Kunming are in a state of subsidence and severely subsiding regions are concentrated in Guandu and Xishan, where the cumulative subsidence reached 210 mm and 98 mm during the period of 2007–2016, respectively. Comparisons of ground deformation between InSAR and leveling observations indicate that RMSE values are about 4.5 mm for the L-band and 3.7 mm for the X-band, demonstrating the reliability of our observations.

(2) A correlation analysis was conducted to investigate the causes of the observed subsidence. The results show that the severe subsidence in Kunming might be the result of soft soil consolidation, building load, and groundwater extraction. Among these factors, soft soil was the foundation for the subsidence, and the building load and groundwater extraction factors accelerated the subsidence process.

This study contributes to the understanding of the spatial-temporal evolution of ground deformation in Kunming. However, it is difficult to clearly describe the mechanism of land subsidence due to a lack of related materials, such as continuous ground-based deformation monitoring data. Therefore, sufficient in situ measurements will be collected in the future to analyze the mechanism of ground deformation in Kunming.

## Figures and Tables

**Table 1:** Parameters of synthetic aperture radar (SAR) data in this study.

No.	Satellite	Orbit Direction	Azimuth Angle (°)	Incidence Angle (°)	Number of SAR Images	Data Period
1	ALOS-1	Ascending	−10	38	20	09/01/2007–07/03/2011
2	COSMO-SkyMed	Descending	10	29	40	13/06/2011–06/01/2016
3	Sentinel-1A	Ascending	−12	39	31	23/01/2015–17/02/2017
4	Sentinel-1A	Descending	11	39	60	25/05/2015–01/09/2019

## References

[1] Fu B.C., Huang Y., Li Q.S., Fu J.Q. (2000). Research of Cause and Classification of the Shallow Layer Soft Soil for Kunming Basin. J. Kunming Univ. Sci. Technol..

[2] Lu N., Hernandez A.J., Ramsey R.D. (2015). Land cover dynamics monitoring with Landsat data in Kunming, China: A cost-effective sampling and modelling scheme using Google Earth imagery and random forests. Geocarto Int..

[3] Hu R.L., Yue Z.Q., Wang L.U., Wang S.J. (2004). Review on current status and challenging issues of land subsidence in China. Eng. Geol..

[4] Yang Y., Huang X.M., Zhang Q., Zhu W. (2017). Investigation Report of Surface Cover and Ground Deformation of Kunming, China (In Chinese).

[5] Youxin J.C., Ruiqi H.Y. (2001). Development Process and Characteristics of Land Subsidence in Kunming. J. Seismol. Res..

[6] Chuandong X., Shucheng T., Feng L. (2001). Clay minerals in Quaternary clayey soil and its relation to the land subsidence in Kunming basin area. Acta Petrologica et Mineralogica.

[7] Chuandong X., Xing L., Baozhu L. (2004). Mechanism analysis of land subsidence in Kunming city area. Chin. J. Geol. Hazard Control.

[8] Yu C., Li Z., Chen J., Hu J.C. (2018). Small magnitude co-seismic deformation of the 2017 Mw 6.4 Nyingchi earthquake revealed by InSAR measurements with atmospheric correction. Remote Sens..

[9] Jo M.J., Jung H.S., Won J.S. (2015). Detecting the source location of recent summit inflation via three-dimensional InSAR observation of Kīlauea volcano. Remote Sens..

[10] Anantrasirichai N., Biggs J., Albino F., Bull D. (2019). A deep learning approach to detecting volcano deformation from satellite imagery using synthetic datasets. Remote Sens. Environ..

[11] Valade S., Ley A., Massimetti F., D’Hondt O., Laiolo M., Coppola D., Loibl D., Hellwich O., Walter T.R. (2019). Towards Global Volcano Monitoring Using Multisensor Sentinel Missions and Artificial Intelligence: The MOUNTS Monitoring System. Remote Sens..

[12] Yu H., Lee H., Cao N., Lan Y. (2019). Optimal baseline design for multibaseline InSAR phase unwrapping. IEEE Trans. Geosci. Remote Sens..

[13] Prébet R., Yan Y., Jauvin M., Trouvé E. (2019). A Data-Adaptive EOF-Based Method for Displacement Signal Retrieval from InSAR Displacement Measurement Time Series for Decorrelating Targets. IEEE Trans. Geosci. Remote Sens..

[14] Wnuk K., Walton G., Zhou W. (2019). Four-dimensional filtering of InSAR persistent scatterers elucidates subsidence induced by tunnel excavation in the Sri Lankan highlands. J. Appl. Remote Sens..

[15] Zou W., Chen L. (2019). Determination of Optimum Tie Point Interval for SAR Image Coregistration by Decomposing Autocorrelation Coefficient. IEEE Trans. Geosci. Remote Sens..

[16] Li Z., Cao Y., Wei J., Duan M., Wu L., Hou J., Zhu J. (2019). Time-series InSAR ground deformation monitoring: Atmospheric delay modeling and estimating. Earth-Sci. Rev..

[17] Wu S., Zhang L., Ding X., Perissin D. (2018). Pixel-Wise MTInSAR Estimator for Integration of Coherent Point Selection and Unwrapped Phase Vector Recovery. IEEE Trans. Geosci. Remote Sens..

[18] Zhang Q., Zhu W., Ding X., Zhao C., Yang C., Qu W. (2014). Two-dimensional deformation monitoring over Qingxu (China) by integrating C-, L-and X-bands SAR images. Remote Sens. Lett..

[19] Haghighi M.H., Motagh M. (2019). Ground surface response to continuous compaction of aquifer system in Tehran, Iran: Results from a long-term multi-sensor InSAR analysis. Remote Sens. Environ..

[20] Peng M., Zhao C., Zhang Q., Lu Z., Li Z. (2019). Research on Spatiotemporal Land Deformation (2012–2018) over Xi’an, China, with Multi-Sensor SAR Datasets. Remote Sens..

[21] Sarychikhina O., Glowacka E., Robles B. (2018). Multi-sensor DInSAR applied to the spatiotemporal evolution analysis of ground surface deformation in Cerro Prieto basin, Baja California, Mexico, for the 1993–2014 period. Nat. Hazards.

[22] Aimaiti Y., Yamazaki F., Liu W. (2018). Multi-sensor InSAR analysis of progressive land subsidence over the Coastal City of Urayasu, Japan. Remote Sens..

[23] Solari L., Del M., Bianchini S., Ciampalini A., Ezquerro P., Montalti R., Raspini F., Moretti S. (2018). From ERS 1/2 to Sentinel-1: Subsidence monitoring in Italy in the last two decades. Front. Earth. Sci..

[24] Aslan G., Cakır Z., Ergintav S., Lasserre C., Renard F. (2018). Analysis of secular ground motions in istanbul from a long-term insar time-series (1992–2017). Remote Sens..

[25] Rimba A.B., Osawa T., Parwata I.N.S., As-syakur A.R., Kasim F., Astarini I.A. (2018). Physical assessment of coastal vulnerability under enhanced land subsidence in Semarang, Indonesia, using multi-sensor satellite data. Adv. Space Res..

[26] Fuhrmann T., Garthwaite M.C. (2019). Resolving Three-Dimensional Surface Motion with InSAR: Constraints from Multi-Geometry Data Fusion. Remote Sens..

[27] Zheng M., Deng K., Fan H., Du S., Zou H. (2019). Monitoring and analysis of mining 3D time-series deformation based on multi-track SAR data. Int. J. Remote Sens..

[28] Wang Z., Zhang R., Wang X., Liu G. (2018). Retrieving three-dimensional co-seismic deformation of the 2017 MW7. 3 Iraq earthquake by multi-sensor SAR images. Remote Sens..

[29] Zhao Q., Ma G., Wang Q., Yang T., Liu M., Gao W., Falabella F., Mastro P., Pepe A. (2019). Generation of long-term InSAR ground displacement time-series through a novel multi-sensor data merging technique: The case study of the Shanghai coastal area. ISPRS J. Photogram. Remote Sens..

[30] Chen X., Zhang Y. (2017). Impacts of urban surface characteristics on spatiotemporal pattern of land surface temperature in Kunming of China. Sustain Cities Soc..

[31] Fan Y.H., Li W.C. (2006). Geological characteristics of the Pulang porphyry copper deposit, Yunnan. Geol. China.

[32] Zebker H.A., Villasenor J. (1992). Decorrelation in interferometric radar echoes. IEEE Trans. Geosci. Remote Sens..

[33] Ferretti A. (2000). Nonlinear subsidence rate estimation using permanent scatters in differential SAR interferometry. IEEE Trans. Geosci. Remote Sens..

[34] Baran I., Stewart M.P., Kampes B.M., Perski Z., Lilly P. (2003). A modification to the Goldstein radar interferogram filter. IEEE Trans. Geosci. Remote Sens..

[35] Costantini M. (1998). A novel phase unwrapping method based on network programming. IEEE Trans. Geosci. Remote Sens..

[36] Zhang L., Ding X., Lu Z., Jung H.S., Hu J., Feng G. (2013). A novel multitemporal InSAR model for joint estimation of deformation rates and orbital errors. IEEE Trans. Geosci. Remote Sens..

[37] Schulz K., Boldt M., Even M. (2012). Generalization of the CoVAmCoh analysis for the interpretation of arbitrary InSAR images. IEEE Int. Geosci. Remote Sens. Symp..

[38] Zhu W., Zhang Q., Ding X., Zhao C., Yang C., Qu F., Qu W. (2014). Landslide monitoring by combining of CR-InSAR and GPS techniques. Adv. Space Res..

[39] Meckel T.A., ten Brink U.S., Williams S.J. (2006). Current subsidence rates due to compaction of Holocene sediments in southern Louisiana. Geophys. Res. Lett..

[40] Bonì R., Bosino A., Meisina C., Novellino A., Bateson L., McCormack H. (2018). A methodology to detect and characterize uplift phenomena in urban areas using Sentinel-1 data. Remote Sens..

[41] Tang Y.Q., Cui Z.D., Wang J.X., Lu C., Yan X.X. (2008). Model test study of land subsidence caused by high-rise building group in Shanghai. Bull. Een. Geol. Environ..

[42] Franzius J.N., Potts D.M., Addenbrooke T.I., Burland J.B. (2004). The influence of building weight on tunneling-induced ground and building deformation. Soils Found..

[43] Yu B., Liu H., Wu J., Hu Y., Zhang L. (2010). Automated derivation of urban building density information using airborne LiDAR data and object-based method. Landsc. Urban Plan..

[44] Li Y., Zhang N. (2017). Assessment of Groundwater Overdraft Zones in Kunming Basin. J. Yangtze River Sci. Res. Inst..

[45] Holzer T.L., Johnson A.I. (1985). Land subsidence caused by ground water withdrawal in urban areas. GeoJournal.

[46] Woldai T., Oppliger G., Taranik J. (2009). Monitoring dewatering induced subsidence and fault reactivation using interferometric synthetic aperture radar. Int. J. Remote Sens..

